# Increased Risk of *Toxoplasma gondii* Infection in Patients with Colorectal Cancer in Eastern China: Seroprevalence, Risk Factors, and a Case–Control Study

**DOI:** 10.1155/2020/2539482

**Published:** 2020-10-06

**Authors:** Yang Yu, Dong Guo, Tingting Qu, Shuchao Zhao, Chang Xu, Longlong Wang, Zhongjun Wang, Haiyang Fu, Xiangyan Zhang, Na Zhou

**Affiliations:** ^1^Department of Clinical Laboratory, The Affiliated Hospital of Qingdao University, Qingdao, China; ^2^Department of Gastrointestinal Surgery, The Affiliated Hospital of Qingdao University, Qingdao, China; ^3^Department of Pathology, The Affiliated Hospital of Qingdao University, Qingdao, China; ^4^Department of oncology, The Affiliated Hospital of Qingdao University, Qingdao, China

## Abstract

The aim of this study was to explore the epidemiology of *Toxoplasma gondii* infection in patients with colorectal cancer (CRC) in eastern China. Therefore, 287 primary CRC patients and 287 age-matched healthy control subjects were recruited to estimate the seroprevalence of *T. gondii* and identify the risk factors of infection. Enzyme-linked immunoassays were used to test for anti-*T. gondii* immunoglobulin G (IgG) and IgM antibodies. Forty-six (16%) samples were positive for anti-*T. gondii* IgG antibodies in patients with CRC, compared with 26 (9.1%) in the healthy controls, a significant difference (*P* = 0.007). By contrast, eight (2.8%) patients tested positive for *T. gondii* IgM antibodies, compared with three (1.1%) in the controls, a difference that was not significant (*P* = 0.13). Multivariable backward stepwise logistic regression analysis revealed that a rural residence (OR 2.83; 95% CI 1.15–7.01; *P* = 0.024) and treatment with chemotherapy (OR 2.16; 95% CI 1.02–4.57; *P* = 0.045) were risk factors for *T. gondii* infection in patients with CRC. Thus, *T. gondii* infection is serious in patients with CRC, and a rural residence and treatment with chemotherapy are independent risk factors for infection by this parasite. Therefore, medical professionals should be aware of this pathogen in patients with CRC, and the causes of *T. gondii* infection in these patients need to be explored further.

## 1. Introduction

Colorectal cancer (CRC) is one of the most common malignancies, with the morbidity increasing steadily in recent years [1]. However, the outcome of CRC patients has improved substantially because of anticancer treatments such as surgery, chemotherapy, and targeted treatment [2]. The most common adverse effects that lead to death in malignancy patients immunocompromisation by tumors and antitumor treatments are complications associated with infection, such as those caused by bacteria and viruses [3, 4]. Parasites, especially those that cause opportunistic infections, have received relatively little attention in malignancy patients.

*Toxoplasma gondii* is one of the most common parasites to cause opportunistic infections, and nearly one-third of humans suffer from chronic infection worldwide [5]. The human immune system can prevent the propagation of *T. gondii*, and thus, an acute acquired infection is generally self-limiting and asymptomatic in healthy humans [6]. However, in immunocompromised patients, such as those with a tumor, *T. gondii* may cause a serious, life-threatening infection. Several studies demonstrate a latent relationship between toxoplasmosis and malignancy, including with brain and oral cancers [7–9]. In recent studies, patients with hematologic malignancies had higher seroprevalence of *T. gondii* antibodies [10, 11]. Moreover, in Iran, *T*. *gondii* DNA was detected in formalin-fixed, paraffin-embedded breast cancer tissue [12].

Cats are the only definitive host, and the oocysts they shed can be viable in soil for many years. Humans can develop toxoplasmosis when they ingest oocysts shed by cats or tissue cysts in other mammals [4]. Many factors are associated with toxoplasmosis in cancer patients, including contact with pigs or cats, consumption of raw or undercooked meat, and exposure to soil [9, 10, 13]. Moreover, histories of blood transfusion and chemotherapy are also risk factors for *T*. *gondii* infection in patients with malignancy [9, 14]. However, in patients with CRC, data on *T*. *gondii* infection are rare, and as a result, the prevalence of *T*. *gondii* infection and the associated risk factors in patients with CRC are unclear. Thus, this study was conducted to investigate the seroprevalence of *T*. *gondii* in patients with CRC and the association between *T*. *gondii* infection and different risk factors.

## 2. Methods

### 2.1. Subjects

Serum samples were collected from 287 primary CRC patients who presented to the Affiliated Hospital of Qingdao University (Qingdao, China) for diagnosis and treatment from May 2016 to June 2019. Patients who had received intravenous immunoglobulin therapy or immunotherapy before blood collection were excluded. The ages of the patients were from 33 to 87 years. The control subjects were 287 persons who participated in health screenings at the Affiliated Hospital of Qingdao University that were recruited and matched with CRC patients by age, gender, and location of residence.

The Ethics Committee of the Affiliated Hospital of Qingdao University approved the study (No. 2016-017-29), and all patients/guardians signed informed consent.

### 2.2. Sample Collection

Approximately, 5 ml of venous blood was drawn from the participants. The blood samples were centrifuged at 3,000 rpm for 10 min. Then, sera were collected in 1.5 ml Eppendorf tubes and stored at −80°C until tested for *T*. *gondii* antibodies.

### 2.3. Sociodemographic and Clinical Data

Sociodemographic data including age, sex, residence location, and occupation were obtained from all participants. A structured questionnaire was used to collect data on lifestyle, including any history of contact with cats, pigs, or dogs kept at home, consumption of raw or undercooked meat or vegetables and fruits, exposure to soil, and source of drinking water [7]. Clinical data that included primary locations of tumors, tumor diameter, TNM stage, histological characteristics, and histories of blood transfusion and chemotherapy were obtained from the medical records and pathological reports of the patients.

### 2.4. Serological Assay

Anti-*T*. *gondii* immunoglobulin G (IgG) and IgM antibodies were tested by using commercially available enzyme-linked immunosorbent assay kits (Kanghua Bio, Inc., China), following the manufacturer's instructions. Briefly, sera diluted 1 : 400 were incubated in a *T*. *gondii* antigen-coated 96-well plate at 37°C for 20 min, followed by washing three times with distilled water, and then, 50 *μ*l of HRP conjugate enzyme was added to each well. After washing five times, “A” (50 *μ*l) and “B” (30 *μ*l) solutions were added into the wells and incubated at 37°C for 10 min. The optical density (OD) was read at 450 nm using an automated microplate reader (Infinit f200, Tecan, Australia) and corrected with blank controls. The cutoff value was calculated as 2.1 times the mean negative control OD, and results equal to or greater than the cutoff value were considered positive. Positive and negative serum controls were included in every plate. The samples from patients with CRC and control subjects were randomly mixed.

### 2.5. Statistics

Statistical analyses were conducted using the statistical software SPSS 19.0. In the single variable analysis, a chi-squared test or Fisher exact test was used to analyze the antibody seroprevalence for various variables. The independent risk factors associated with *T*. *gondii* infection were identified by a multivariable backward stepwise logistic regression analysis. The adjusted odds ratio (OR) and 95% confidence interval (CI) were calculated to identify the effect size of risk factors. Results with *P* < 0.05 were considered as statistically significant.

## 3. Results

The overall seroprevalence of *T*. *gondii* antibodies was 48/287 (16.7%) in CRC patients and 26/287 (9.1%) in healthy controls, a significant difference (*P* = 0.006). The seroprevalence of anti-*T*. *gondii* IgG antibodies was significantly different (*P* = 0.007) between CRC patients (46/287, 16.0%) and healthy controls (26/287, 9.1%). By contrast, eight (2.8%) patients tested positive for *T*. *gondii* IgM antibodies, compared with three (1.1%) controls, a difference that was not significant (*P* = 0.13). The single variable analysis showed that *T*. *gondii* seroprevalence was associated with residence in a rural area, an advanced tumor stage, and chemotherapy treatment. A higher seroprevalence of *T. gondii* antibodies was found in patients ≤50 years old (22.58%) than in those 51 to 69 (15.98%) or ≥70 years old (16.13%), although the difference was not significant (*P* = 0.65). In addition, patients with a left-side colon tumor or those with a nonmucin tumor had a higher *T*. *gondii* seroprevalence, although the differences were not significant. The sociodemographic and clinical characteristics of the CRC patients and the healthy controls are shown in Table 1.

The multivariable backward stepwise logistic regression analysis revealed that residence in a rural area (OR 2.83; 95% CI 1.15–7.01; *P* = 0.024) and chemotherapy treatment (OR 2.16; 95% CI 1.02–4.57; *P* = 0.045) were significantly associated with *T*. *gondii* infection (Table 2).

## 4. Discussion

Genetic factors and physical and chemical factors are responsible for the development of CRC. Although some reports indicate a possible association between *T*. *gondii* infection and malignancies [9, 15, 16], the infection status of *T*. *gondii* in patients with CRC remains unclear in eastern China.

In this study, the *T*. *gondii* seroprevalence was significantly higher in patients with CRC (16.7%, 48/287) than in control subjects (9.1%%, 26/287) (*P* = 0.006). The *T*. *gondii* seroprevalence is higher than the 8.4% reported among 119 CRC patients in Anhui, China [17], but lower than the 21.7% reported in Hainan, China [18]. Compared with China, *T*. *gondii* seroprevalence is higher in the Middle East and Egypt, where it reaches 50% in patients with CRC [19, 20]. Several factors may be responsible for these differences, such as the sample size and the age distribution and race of patients, as well as diet and environmental factors. In addition, compared with healthy controls, patients with CRC had a higher seroprevalence of *T*. *gondii* IgG antibodies (16.0% vs. 9.1%; *P* = 0.007), although the seroprevalence of *T. gondii* IgM antibodies was not significantly different between the two groups (2.8% vs. 1.1%; *P* = 0.13). Thus, patients with malignancies more commonly harbored *T*. *gondii* infection. However, whether cancer opens a door for toxoplasmosis or toxoplasmosis promotes the development of cancer remains equivocal. *Toxoplasma gondii* has some rhoptry-secreted kinases that promote intracellular infection and inhibit the innate immune response in mammals [21, 22]. *Toxoplasma gondii* may also promote the development of brain cancer by transforming the expression of host microRNAs [23]. Moreover, in malignant tumors, there are defective both humoral and innate immune responses, and patients struggle to resist intracellular pathogen infections and are inclined to develop toxoplasmosis [22]. Thus, both toxoplasmosis and cancer are interacting. This study showed clearly that *T*. *gondii* is associated with CRC; however, the reasons for the association remain unclear, and the effects of *T*. *gondii* infection on CRC patients need to be studied further.

The rate of *T*. *gondii* infection increases with age [24, 25] and is affected by sex [16]. These associations can be explained because the possibility of infection increases with age and depends on type of occupation, often sex-related, with an opportunistic pathogen such as *T*. *gondii*. However in patients with malignancy, *T*. *gondii* is inclined to infect younger patients [10]. The reason for this association is that patients with malignancy are immune-defective, and young patients may have immune systems too frail to control opportunistic infections [7, 10]. In the present study, the *T*. *gondii* seroprevalence was highest in patients ≤50 years old, although the increase was not significant. Therefore, further studies based on larger sample sizes are needed to confirm the association between *T*. *gondii* infection and age of CRC patients.

Previous reports indicate that contact with pigs and cats, consumption of raw or undercooked meat, and exposure to soil are risk factors for *T*. *gondii* infection in cancer patients [7, 9]. In the present study, the multivariate analysis showed that patients residing in a rural area had higher seroprevalence than those residing in an urban area. Oocysts are shed by felines infected with *T*. *gondii*, and humans got infected via ingestion of oocysts. Therefore, contact with felines and drinking water contaminated with feline feces can increase the risk of *T*. *gondii* infection [26]. Rural areas are typically less developed with poor sanitation. In addition, most people who live in rural areas are farmers and are exposed to feces more frequently, thereby increasing the risk of new infection by the intracellular parasite. Although the patients that resided in rural areas had higher risk of *T*. *gondii* infection, this problem has received little attention. Therefore, there is an urgent need to appeal to the general public to take action and reduce *T*. *gondii* infection in people residing in rural areas. Additionally, in the patients from rural areas with CRC, it is essential to monitor for early signs of toxoplasmosis and to take steps to prevent *T*. *gondii* infection.

Chemotherapy is the main treatment for patients with advanced CRC, although it may cause immune system dysfunction and lead to failure of cancer therapy [27]. In fact, with chemotherapy, patients may be more susceptible to toxoplasmosis [15, 28]. Patients with malignant tumors have defective immunity, and when treated with chemotherapy, their immune systems weaken further and then fail to prevent *T*. *gondii* infection [7]. Paclitaxel and 5-fluorouracil (5-FU) are the most commonly used chemotherapy drugs for CRC. Paclitaxel activates ERK through the toll-like receptor 4-myeloid differentiation gene 88 signaling pathway, whereas 5-FU leads to the misincorporation of fluoronucleotides into RNA and DNA and inhibits the nucleotide synthetic enzyme thymidylate synthase. 5-FU-induced activation of MAPK activity is associated with increased production of IL-1, IL-6, and TNF, which may increase inflammation responses. In this study, the single variable analysis showed that patients with an advanced stage of tumor were more likely to be infected with *T*. *gondii*. Therefore, an advanced tumor stage and chemotherapy may interact in affecting the development of toxoplasmosis. Moreover, similar to other intracellular pathogens, *T*. *gondii* intracellular tachyzoites can disseminate into all organs and may disturb cellular barriers and cause irregularity in host gene expression. Thus, people with CRC may be susceptible to *T*. *gondii* and develop toxoplasmosis.

There were some limitations associated with this study. First, the dataset was relatively small and therefore did not reflect the entire Chinese population. Second, antibody-based assays may contribute to false positivity of the results as confirmatory tests based on antigen detection have not been employed in the study, so, the influence of this management for antibody seroprevalence was unclear. Third, the sera of the donors were not collected, so the positives for *T*. *gondii* antibodies caused by donor-derived antibodies were uncertain.

## 5. Conclusions

The present study revealed that *T*. *gondii* infection is serious in patients with CRC and that residence in a rural area and treatment with chemotherapy are independent risk factors for this parasitic infection. Thus, doctors should be careful with this pathogen in patients with CRC, and the causes of *T*. *gondii* infection in those patients need to be explored further.

## Figures and Tables

**Table 1:** Seroprevalence of *Toxoplasma gondii* infection in colorectal cancer (CRC) patients and control subjects in eastern China.

	Patients with CRC (*n* = 287)				Controls (*n* = 287)			
Characteristic	Prevalence of *T. gondii* infection				Prevalence of *T. gondii* infection			
	No. tested	No. positive	%	*P*	No. tested	No. positive	%	*P*
Age (years)								
≤50	31	7	22.58%	0.65	28	2	7.14%	0.93
51 to 69	194	31	15.98%		152	14	9.21%	
≥70	62	10	16.13%		107	10	9.35%	
Sex								
Male	156	23	14.74%	0.33	173	16	9.25%	0.89
Female	131	25	19.08%		114	10	8.77%	
Residence area								
Urban	77	6	7.79%	0.014	122	6	4.92%	0.036
Rural	210	42	20.00%		165	20	12.12%	
Contact with cats								
Yes	109	13	11.93%	0.09	127	13	10.24%	0.54
No	178	35	19.66%		160	13	8.13%	
Contact with dogs								
Yes	47	8	17.02%	0.95	80	5	6.25%	
No	240	40	16.67%		207	21	10.14%	0.31
Contact with swine								
Yes	40	7	17.50%	0.89	47	4	8.51%	0.88
No	247	41	16.60%		240	22	9.17%	
Consumption of raw/undercooked meat								
Yes	61	5	8.20%	0.44	42	2	4.76%	0.39
No	226	43	19.03%		245	24	9.80%	
Consumption of raw vegetables								
Yes	198	32	16.16%	0.71	226	22	9.73%	0.44
No	89	16	17.98%		61	4	6.56%	
Exposure to soil								
Yes	162	28	17.28%	0.77	172	13	7.56%	0.28
No	125	20	16.00%		115	13	11.30%	
Source of drinking water								
Tap	203	11	5.42%	0.29	207	17	8.21%	0.42
Well + river	84	37	44.05%		80	9	11.25%	
Volunteer's occupation								
Farmer	135	24	17.78%	0.65	153	17	11.11%	0.2
Worker	152	24	15.79%		134	9	6.72%	
Tumor location								
Left side colon	74	16	21.62%	0.29				
Right side colon	53	10	18.87%					
Rectum	160	22	13.75%					
Tumor diameter								
≤5 cm	199	33	16.58%	0.92				
>5 cm	88	15	17.05%					
Tumor stage								
I + II	169	22	13.02%	0.044				
III + IV	118	26	22.03%					
Mucin production								
With	36	4	11.11%	0.34				
Without	251	44	17.53%					
Blood transfusion history								
Yes	23	4	17.39%	0.93				
No	264	44	16.67%					
Chemotherapy history								
Yes	188	38	20.21%	0.029				
No	99	10	10.10%					

**Table 2:** Multivariable analysis of patients with colorectal cancer and healthy controls and the association of characteristics with *Toxoplasma gondii* infection.

Characteristic^a^	Adjusted odds ratio^b^	95% CI^c^	*P*
Residence area	2.83	1.15–7.01	0.024
Chemotherapy history	2.16	1.02–4.57	0.045
Contact with cats	0.65	0.33–1.29	0.21
Contact with swine	1.99	0.80–4.93	0.34

^a^ ^b^ ^c^Multivariable backward stepwise logistic regression analysis. Adjusted by age. Confidence interval.

## Data Availability

Requests for access to individual subject data may be made to Na Zhou; please send an email to zhou_na_love@126.com.

## References

[1] Siegel R. L., Miller K. D., Jemal A. (2019). Cancer statistics, 2019. *CA: a Cancer Journal for Clinicians*.

[2] Bray F., Ferlay J., Soerjomataram I., Siegel R. L., Torre L. A., Jemal A. (2018). Global cancer statistics 2018: GLOBOCAN estimates of incidence and mortality worldwide for 36 cancers in 185 countries. *CA: a Cancer Journal for Clinicians*.

[3] Vehreschild M. J., Hamprecht A., Peterson L. (2014). A multicentre cohort study on colonization and infection with ESBL-producing Enterobacteriaceae in high-risk patients with haematological malignancies. *The Journal of Antimicrobial Chemotherapy*.

[4] Razavi-Shearer D., Gamkrelidze I., Nguyen M. H. (2018). Global prevalence, treatment, and prevention of hepatitis B virus infection in 2016: a modelling study. *The Lancet Gastroenterology & Hepatology*.

[5] Elmore S. A., Jones J. L., Conrad P. A., Patton S., Lindsay D. S., Dubey J. P. (2010). *Toxoplasma gondii*: epidemiology, feline clinical aspects, and prevention. *Trends in Parasitology*.

[6] Shaw M. H., Reimer T., Sánchezvaldepeñas C. (2009). T cell-intrinsic role of Nod2 in promoting type 1 immunity to *Toxoplasma gondii*. *Nature Immunology*.

[7] Cong W., Liu G. H., Meng Q. F. (2015). *Toxoplasma gondii* infection in cancer patients: prevalence, risk factors, genotypes and association with clinical diagnosis. *Cancer Letters*.

[8] Thomas F., Lafferty K. D., Brodeur J., Elguero E., Gauthier-Clerc M., Missé D. (2012). Incidence of adult brain cancers is higher in countries where the protozoan parasiteToxoplasma gondiiis common. *Biology Letters*.

[9] Zhou N., Zhang X. Y., Li Y. X., Wang L., Wang L. L., Cong W. (2018). Seroprevalence and risk factors of *Toxoplasma gondii* infection in oral cancer patients in China: a case-control prospective study. *Epidemiology and infection*.

[10] Zhou N., Fu H., Wang Z. (2019). Seroprevalence and risk factors of *Toxoplasma gondii* infection in children with leukemia in Shandong Province, Eastern China: a case-control prospective study. *PeerJ*.

[11] Kalantari N., Rezanejad J., Tamadoni A., Ghaffari S., Alipour J., Bayani M. (2018). Association between *Toxoplasma gondii* exposure and paediatrics haematological malignancies: a case-control study. *Epidemiology and infection*.

[12] N K., Z A. D., S S. (2017). *Toxoplasma gondii* detection of DNA in malignant breast tissues in breast cancer patients. *International journal of molecular and cellular medicine*.

[13] Alvarado-Esquivel C., Liesenfeld O., Torres-Castorena A. (2010). Seroepidemiology of *Toxoplasma gondii* infection in patients with vision and hearing impairments, cancer, HIV, or undergoing hemodialysis in Durango, Mexico. *The Journal of parasitology*.

[14] Scerra S., Coignard-Biehler H., Lanternier F. (2013). Disseminated toxoplasmosis in non-allografted patients with hematologic malignancies: report of two cases and literature review. *European journal of clinical microbiology & infectious diseases : official publication of the European Society of Clinical Microbiology*.

[15] Ali M. I., Abd El Wahab W. M., Hamdy D. A., Hassan A. (2019). *Toxoplasma gondii* in cancer patients receiving chemotherapy: seroprevalence and interferon gamma level. *Journal of parasitic diseases : official organ of the Indian Society for Parasitology*.

[16] Anvari D., Sharif M., Sarvi S. (2019). Seroprevalence of *Toxoplasma gondii* infection in cancer patients: a systematic review and meta-analysis. *Microbial pathogenesis*.

[17] Wang L., He L. Y., Meng D. D. (2015). Seroprevalence and genetic characterization of *Toxoplasma gondii* in cancer patients in Anhui Province, Eastern China. *Parasites & vectors*.

[18] Chun-Yun L. (2019). Investigation on infections among patients with malignant tumors of the digestive tract in Hainan Province. *Zhongguo xue xi chong bing fang zhi za zhi = Chinese journal of schistosomiasis control*.

[19] Molan A. L., Rasheed E. H. (2016). Study the possible link between toxoplasmosis and different kinds of cancer in Iraq. *American Journal of Life Ence Researches*.

[20] Abdel Malek R., Wassef R., Rizk E., Sabry H., Tadros N., Boghdady A. (2018). Toxoplasmosis an overlooked disease: seroprevalence in cancer patients. *Asian Pacific Journal of Cancer Prevention : APJCP*.

[21] Lorenzi H., Khan A., Behnke M. S. (2016). Local admixture of amplified and diversified secreted pathogenesis determinants shapes mosaic *Toxoplasma gondii* genomes. *Nature communications*.

[22] Li J. X., He J. J., Elsheikha H. M. (2019). *Toxoplasma gondii* ROP17 inhibits the innate immune response of HEK293T cells to promote its survival. *Parasitology Research*.

[23] Thirugnanam S., Rout N., Gnanasekar M. (2013). Possible role of *Toxoplasma gondii* in brain cancer through modulation of host microRNAs. *Infectious Agents & Cancer*.

[24] Imam A., Al-Anzi F., Al-Ghasham M., Al-Suraikh M., Al-Yahya A., Rasheed Z. (2017). Serologic evidence of *Toxoplasma gondii* infection among cancer patients. A prospective study from Qassim region, Saudi Arabia. *Saudi Medical Journal*.

[25] Nowakowska D., Wujcicka W., Sobala W., Śpiewak E., Gaj Z., Wilczyński J. (2014). Age-associated prevalence ofToxoplasma gondiiin 8281 pregnant women in Poland between 2004 and 2012. *Epidemiology & Infection*.

[26] Tian A. L., LI G. X., Elsheikha H. M. (2017). Seroepidemiology of *Toxoplasma gondii* infection in patients with liver disease in eastern China. *Epidemiology & Infection*.

[27] Vyas D., Laput G., Vyas A. K. (2014). Chemotherapy-enhanced inflammation may lead to the failure of therapy and metastasis. *Oncology Targets and therapy*.

[28] Duan Y., Zhi Y., Liu Y. (2019). *Toxoplasma* gondiiinfection in children with lymphoma in Eastern China: seroprevalence, risk factors and case-control studies. *Epidemiology and infection*.

